# An Environmental‐Inert and Highly Self‐Healable Elastomer Obtained via Double‐Terminal Aromatic Disulfide Design and Zwitterionic Crosslinked Network for Use as a Triboelectric Nanogenerator

**DOI:** 10.1002/advs.202202815

**Published:** 2022-12-01

**Authors:** Syun‐Hong Chou, Hong‐Wei Lu, Ta‐Chung Liu, Yi‐Ting Chen, Yen‐Lin Fu, Yung‐Hsin Shieh, Ying‐Chih Lai, San‐Yuan Chen

**Affiliations:** ^1^ Department of Materials Science and Engineering National Yang Ming Chiao Tung University Hsinchu 30010 Taiwan; ^2^ Department of Materials Science and Engineering National Chung Hsing University Taichung 40227 Taiwan; ^3^ Department of Biomedical Engineering National Yang Ming Chiao Tung University Taipei 112304 Taiwan; ^4^ Department of Materials Science and Engineering National Tsing Hua University Hsinchu 300044 Taiwan; ^5^ Innovation and Development Center of Sustainable Agriculture i‐Center for Advanced Science and Technology National Chung Hsing University Taichung 40227 Taiwan; ^6^ Graduate Institute of Biomedical Science China Medical University Taichung City 406040 Taiwan; ^7^ Frontier Research Centre on Fundamental and Applied Sciences of Matters National Tsing Hua University Hsinchu 300044 Taiwan; ^8^ School of Dentistry College of Dental Medicine Kaohsiung Medical University Kaohsiung City 80708 Taiwan

**Keywords:** autonomous self‐healable materials, harsh environment, human–device interfaces, power sources, triboelectric nanogenerators

## Abstract

Due to the ongoing development of portable/mobile electronics, sources to power have received widespread attention. Compared to chemical batteries as power sources, triboelectric nanogenerators (TENGs) possess lots of advantages, including the ability to harvest energy via human motions, flexible structures, environment‐friendliness, and long‐life characteristics. Although many self‐healable TENGs are reported, the achievement of a muscle‐like elasticity and the ability to recover from inevitable damage under extreme conditions (such as a high/low temperature and/or humidity) remain a challenge. Herein, a “double‐terminal aromatic disulfide” on a structure with zwitterions as branched chains is reported to engineer the high‐efficient self‐healable elastomer for application in a flexible TENG. The as‐designed material exhibits a repeatable elastic recovery (at 250% elongation) and a self‐healing efficiency with an ultimate tensile stress of 96% over 2 h, representing an improvement on previously reported disulfide‐based elastomers. The elastomer can autonomously recover by 50% even at a subzero temperature of −30 °C within 24 h. The elastomer‐based TENG, as a self‐driven sensor for detecting human behavior, is demonstrated to exhibit stable outputs and self‐healing in the temperature range of −30 to 60 °C, and so is expected to promote the development of self‐powered electronics for next‐generation human–machine communications.

## Graphical abstract

An environmental‐inert and self‐healing triboelectric nanogenerator based on double‐terminal aromatic disulfide bonds and zwitterions not only acts as power generators but also serves as the self‐powered human–machine interface device via harvesting biomechanical energy. Notably, the energy harvester remains the excellent properties at harsh conditions such as subzero regions and the desert.

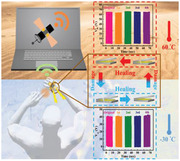

## 1 Introduction

With the rapid evolution of the Internet of Things (IoTs), the development of durable energy devices is highly demanded for autonomous electronics.^[^ ^1^, ^2^, ^3^, ^4^, ^5^, ^6^, ^7^, ^8^ ^]^ However, issues arise in terms of the typical batteries when the IoT devices are in harsh environments.^[^ ^9^, ^10^, ^11^, ^12^ ^]^ For example, batteries are rapidly discharged and even damaged at subzero temperatures or in high humidity/dry climates. Thus, the development of durable energy harvesters that can function in harsh environments is required for emerging IoT devices. Moreover, considering the unpredictable damage that can occur to such energy harvesters, their ability to undergo self‐healing is also crucial to their long‐term usage.^[^ ^13^, ^14^ ^]^

Triboelectric nanogenerators (TENGs), which can convert ambient mechanical energy into electricity, have shown great potential for use in portable devices, wearables, implants, and robotics.^[^ ^2^, ^15^, ^16^, ^17^ ^]^ Their merits include diversity in materials selection, cost‐effective fabrication, and high output performance. As such, several efforts have been made to develop flexible and stretchable TENGs to widen their applications.^[^ ^18^, ^19^, ^20^, ^21^ ^]^ For example, among the state‐of‐the‐art materials reported for use in TENGs, hydrogels have attracted significant attention owing to their structural similarities to biological soft tissues, their intrinsic repair capabilities, and their flexible nature.^[^ ^13^, ^22^, ^23^, ^24^ ^]^ However, hydrogels contain substantial amounts of water, which can lead to ice formation at subzero temperatures. As a result, the hydrogels become brittle and they lose their healing capability under such harsh conditions.^[^ ^25^, ^26^, ^27^ ^]^ Although an environmentally friendly hydrogel‐based TENG with a high flexibility and recyclability has previously been reported,^[^ ^28^ ^]^ this device would not be suitable for use under subzero conditions. Similarly, a multifunctional TENG consisting of a metal‐coordinated polymer was developed for use as a triboelectrically charged layer, which was coupled to a hydrogen‐bonded ionic gel as an electrode; however, the performance of the electrode was severely limited at low temperatures.^[^ ^23^ ^]^

To address the above issues, a number of groups have reported the development of stretch‐matched liquid electrode‐based TENGs that exhibit antifreezing properties; this was achieved by integrating a lithium chloride electrolyte, graphene oxide, and ethylene glycol.^[^ ^29^ ^]^ In addition, a low temperature‐resistant hydrogel has been designed based on the polymerization of an acrylamide monomer in an aqueous solution of hydroxyethyl cellulose to provide an electrode for TENG applications. However, the above devices did not exhibit self‐healing properties following damage at low temperature, and so the combination of both properties for application in subzero temperatures or high humidity/dry climates remains the challenge.^[^ ^13^, ^24^, ^30^, ^31^ ^]^

Herein, we propose a newly designed symmetric double‐terminal aromatic disulfide elastomer (SDDE) for the development of a flexible TENG (SDDE‐TENG) that is environmentally inert and exhibits a prominent self‐healing capability. In this system, the double‐terminal aromatic disulfides construct the primary self‐healing network and enhance the number of repairing active sites via increasing the number of aromatic disulfide bonding units at the two ends of the main chain. Furthermore, the grafted zwitterionic polymers provide multiple intramolecular hydrogen bonds, which establish a strongly crosslinked nature and provide a secondary self‐healing network system. The synergistic interactions between these bonds will be expected to endow the elastomer with an extremely high self‐healing efficiency at room temperature (96% of ultimate tensile stress recovery in 2 h) and a stable elastic recovery (250% elongation of repeatable elastic deformation recovery). Moreover, the obtained SDDE‐TENG system is tested under a range of extreme conditions and following cyclic cutting/scratching damage to determine the stability and reliability. The newly designed SDDE‐TENG with outstanding properties can be utilized as a power supply and a self‐powered sensor for vast emerging fields including flexible electronics, smart interfaces, and robotic skins.

## 2 Results and Discussion

In the system, due to the good thermal stability and efficient metathesis, the aromatic‐disulfide bond‐based structure has the self‐healing ability in the ambient temperature. In addition, the zwitterionic polymer, [2‐(methacryloyloxy) ethyl] dimethyl‐(3‐sulfopropyl) ammonium hydroxide (SBMA), is known for its excellent hydration ability. The presence of zwitterions in the polymer network is able to hinder the ice formation and thereby further retain the self‐healing effect at subzero temperatures owing to their strong hydration.^[^ ^32^ ^]^ This combination of aromatic‐disulfides bond‐based structure and SBMA is expected to solve the above problems of conventional hydrogels.

**Figure 1:**
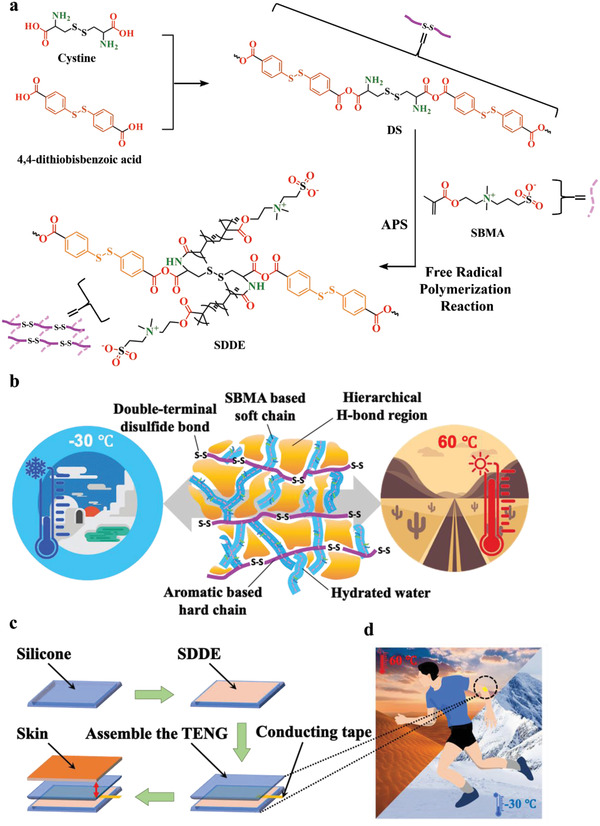
a) Synthetic strategy employed for fabrication of the SDDE electrode of the triboelectric nanogenerator. b) Schematic illustration of the SDDE network and its design concept based on double‐terminal disulfide bonds crosslinked by SBMA zwitterionic side chains. c) Schematic representation showing fabrication of the SDDE‐based triboelectric nanogenerator device. d) Proof‐of‐concept demonstration of a universal human–computer interfacing SDDE‐based triboelectric nanogenerator.

**Figure** **1**a illustrates the synthetic route to the SDDE, and the detailed synthetic procedures are described in the Experimental Section. The chemical structure of the SDDE was confirmed by Fourier transform infrared (FTIR) spectroscopy and X‐ray photoelectron spectroscopy (XPS), as shown in Figures S1 and S2 in the Supporting Information. The designed self‐healing networks of the SDDE elastomer are shown in Figure 1b, wherein it can be seen that the double‐terminal aromatic disulfide‐based structure served as the primary self‐healing system and the hard segment (main chain) in the elastomer. More specifically, methacrylic anhydride was reacted with the amine groups of cystine and the resulting double bond functionalities were covalently bound to SBMA to initiate further free radical polymerization. In this system, the carbonyl and amine groups on the main chain interact with the sulfite groups of the polymerized SBMA (branched chain) to form multiplexed intramolecular hydrogen bonds, generating a crosslinked network and serving as a secondary self‐healing system. Figure 1c illustrates a fabrication process of the SDDE‐TENG, while its potential applications in energy harvesting and human–device interfaces are illustrated in Figure 1d.

**Figure 2:**
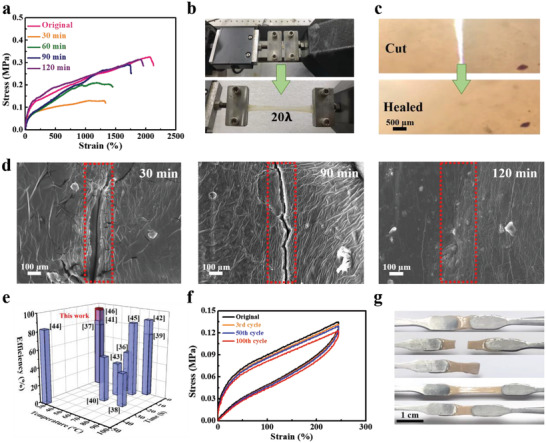
a) Tensile stress–strain profile of the SDDE under ambient conditions with different healing times. b) Photographic images of the SDDE with the original and 20× strain elongations. c) Optical microscopy images of the SDDE from cutting to healing under ambient conditions over 2 h. d) SEM images of the self‐healed SDDE showing the cutting traces taken at times of 30, 90, and 120 min under ambient conditions. e) Comparison of our working SDDE with other disulfide‐based self‐healable materials in terms of the healing efficiency, temperature, and time.^[^ ^36^, ^37^, ^38^, ^39^, ^40^, ^41^, ^42^, ^43^, ^44^, ^45^, ^46^ ^]^ f) Cyclic tensile stress–strain profiles for the SDDE and the self‐healed SDDE with 250% strain (healing time = 2 h). g) Photographic images showing the excellent elastic recoveries of the SDDE and the self‐healed SDDE with 250% tensile strain.

Microtensile tests were performed to evaluate the mechanical properties and self‐healing efficiency of the SDDE at a stretching loading rate of 0.1 mm s^−1^ under ambient conditions. **Figure** **2**a shows the tensile strain–stress profiles for the pristine SDDE and the cut‐and‐healed samples with various healing times ranging from 30 to 120 min. As indicated, the pristine SDDE exhibited a tensile strength of 324 kPa and a tensile strain of 2076%, in addition to a high toughness value of 4.79 MJ m^−3^. Compared to the self‐healing ability of SBMA (Figure S3, Supporting Information), it can be observed the SDDE self‐healing ability is better (96% of ultimate tensile stress recovery in 2 h). The SDDE also displayed an elastomer‐like behavior within 250% strain, and this contrasts to the plastic‐like behavior of a hydrogel, which undergoes permanent deformation (i.e., a nonreversible change in shape in response to force). The high toughness and elastomer‐like behavior of the SDDE were attributed to its intricate crosslinked nature and multiple intramolecular hydrogen bonds. It was also found that the SDDE exhibited a tensile strain of 2000% after 2 h of healing (Figure 2b). As shown in the time‐dependent optical microscopy images of the crack in the bifurcated SDDE (Figure 2c), the cracks clearly vanished after 2 h of healing. In addition, scanning electron microscopy (SEM) observations showed that the cutting traces healed over time under ambient conditions (Figure 2d). In structural terms, this outstanding self‐healing efficiency of the SDDE was attributed to two main characteristics, as follows. First, the double‐terminal disulfide bonding worked as an active repairing unit that boosted self‐healing recovery. Moreover, owing to the low thermoactivation energy barrier in the aromatic‐based structure, the SDDE was able to self‐repair through the reformation of covalent S—S bonds at room temperature. The low thermoactivation energy barrier in the aromatic‐based structure was attributed to delocalization, which resulted in a higher thermal stability.^[^ ^33^, ^34^, ^35^ ^]^ Second, the 3D multiple intermolecular hydrogen bonding networks serve as a secondary repair system to accelerate the self‐healing dynamics. It is worth noting that to the best of our knowledge, the self‐healing efficiency (96%, tensile strength) of our SDDE within 2 h at ambient temperature is the highest reported for any disulfide‐based material (Figure 2a,e).^[^ ^36^, ^37^, ^38^, ^39^, ^40^, ^41^, ^42^, ^43^, ^44^, ^45^, ^46^ ^]^ The elasticity recovery of the SDDE was also evaluated by means of cyclic loading/unloading tests with 250% elongation (Figure 2f). As shown in Figure 2g, the SDDE retained its original viscoelastic behavior (250% elongation) after three loading/unloading cycles, even following self‐healing from bifurcating damage. The definition of viscoelasticity is that elastic deformation will continue after stress and require some finite time for complete recovery after upon load release. As a result, the SDDE needs a while to return to its original shape.^[^ ^47^ ^]^ The tensile tests at the higher stretching rates were shown in Figure S4 in the Supporting Information. It can be observed that the sample with a 50 mm min^−1^ loading speed displayed the highest tensile strength of 0.53 MPa. The reason is that an increasing loading speed reduces the mobility of the polymer, leading to the stiffer.^[^ ^48^ ^]^

**Figure 3:**
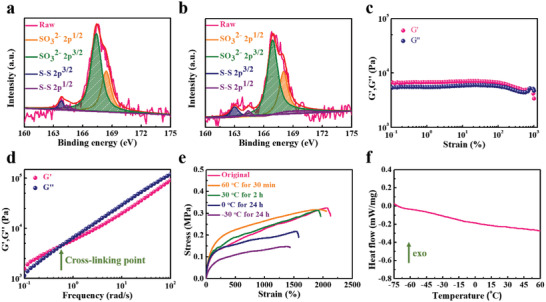
a) High‐resolution XPS spectra of the S 2p region for the SDDE. b) High‐resolution XPS spectra of the S 2p region for the self‐healed SDDE under ambient conditions (healing time = 1 h). c) Strain dependences of *G*′ and *G*′′ for the SDDE. Note that the sweeping frequency was 1 rad s^−1^ under ambient conditions. d) Frequency dependences of *G*′ and *G*′′ for the SDDE. Note that the shear strain corresponded to 5% strain under ambient conditions. e) Tensile stress–strain profile of the SDDE with its maximum recovery at different temperatures. f) Low‐temperature DSC profile of the SDDE. Note that the data were obtained during cycling from −75 to 60 °C at a rate of 5 °C min^−1^.

The self‐healing mechanism of the SDDE was then analyzed by XPS examinations of the surface atomic distribution before and after self‐healing (**Figure** **3**a,b), wherein the integrated areas of the deconvoluted peaks in the high‐resolution XPS spectrum for single‐atom orbits can be used to obtain the respective fractions of the various bond types. Thus, we examined the high‐resolution S 2p spectrum of the SDDE before and after self‐healing, and the fractions for the two different deconvoluted sulfite ions (SO_3_^2−^) and disulfide bonds (S—S) were calculated to be 92.9% and 7.1% before healing and 81.5% and 18.5% after healing, respectively (Table S1, Supporting Information).^[^ ^49^, ^50^ ^]^ The relatively increased fraction of disulfide bonds after healing clearly indicates that the primary self‐healing mechanism for SDDE involves double‐terminal disulfide bonding which is able to migrate to the cleavage surface and heal the crash, since the sulfite ions present in zwitterions are unable to perform repairs.^[^ ^37^ ^]^ In addition, the nonlinear oscillatory shear measurement for the SDDE is shown in Figure 3c, wherein the storage modulus and the loss modulus, denoted as *G*′ and *G*′′, respectively, can be observed. The finding that *G*′ > *G*′′ demonstrates that the SDDE behaves as a stable elastic solid state instead of a viscous liquid state;^[^ ^51^ ^]^ however, it should be noted that both *G*′ and *G*′′ adopted similar constant values in the range of 0.1–93% strain, which indicates a linear elastic regime over this range, and supports the tensile elastic behavior observed in the microforce testing results (i.e., 250% elongation). However, between strains of 93% and 579%, the strain thinning occurred (*G*′ and *G*′′ both decreased), which was attributed to significant deformation of the polymer interchains. Figure 3d shows the frequency‐dependent profile of the nonlinear *G′* and *G*′′ for the SDDE with 5% shear strain. The observation of a crossover point between *G′* and *G*′′ at 0.63 rad s^−1^ indicates that the polymeric network changed from a robust crosslinked and elastic state to a reversible and dynamic liquid state. The presence of this crossover point with a characteristic relaxation time (i.e., 1.59 s) signifies the rapid rearrangement of the SDDE network, which is consistent with the results obtained for healing efficiencies.^[^ ^51^, ^52^ ^]^

We further evaluated the self‐healing efficiencies at different temperatures, as outlined in Figure 3e. More specifically, at a temperature (*T*) of 60 °C, and relative humidity (RH) of 50%, the SDDE exhibited an extremely high healing efficiency of 98% within 30 min owing to the active dynamics of the molecules at high temperatures.^[^ ^53^, ^54^ ^]^ The mechanical properties of the SDDE can be maintained after 50‐time healing at 60 °C (Figure S5, Supporting Information). In addition, upon lowering the temperature to 25 and 0 °C (RH = 50% in both cases), the healing efficiency of the SDDE was 96% after 2 h and 66% after 24 h, respectively. Importantly, at −30 °C, a healing efficiency of 52% after 24 h was achieved. The self‐healing efficiencies at −30 °C with various healing times were evaluated in Figure S6 in the Supporting Information. No significant difference in the self‐healing efficiency has been observed between the samples after 24 h healing and 72 h healing. To further explore the self‐healing nature of the SDDE at low temperatures, low‐temperature differential scanning calorimetry (LT‐DSC) was employed. As shown in Figure 3f, no obvious glass transition point (*T*_g_) was observed between −75 and 0 °C, and this was attributed to the large size of the polymerized SBMA, which is regarded as a substituted group. This increases the disorder of the polymer arrangement and hinders the formation of crystalline structures. Moreover, the zwitterionic groups bound with abundant hydrated water molecules soften the polymer, which also results in limited crystal formation. Notably, a limited endothermic event (i.e., the melting of ice) was observed at ≈0 °C in the DSC profile, indicating that ice formation was suppressed at low temperatures. This was due to strong bonding with zwitterions suppressing the activity of the water molecules, and so ice was unable to form at subzero temperatures. Thus, the disordered polymeric structure and the limited amount of ice formation impart the SDDE with an excellent self‐healing performance at subzero temperatures.

To evaluate the long‐term material stability, the SDDE was exposed to UV light at high temperatures. After 4 weeks of UV light exposure at 60 °C, neither material yellowing nor cracking was observed on the SDDE surface (Figure S7, Supporting Information). The surface atomic composition of the exposed SDDE was then further investigated by XPS, and it was found that the oxygen fraction was similar to that of the pristine unexposed SDDE, thereby indicating that limited oxidation occurred (Figure S8 and Table S2, Supporting Information). In addition, the tensile stress–strain profile and the healing profiles recorded over different healing times were obtained for the exposed SDDEs and were found to be relatively unchanged compared with those of the pristine unexposed SDDE (Figure S9, Supporting Information). The remarkable antiaging performance of the SDDE was therefore attributed to the aromatic disulfide bonding, which serves as an antioxidant to remove free radicals and inhibit further chain‐to‐chain oxidative reactions.

**Figure 4:**
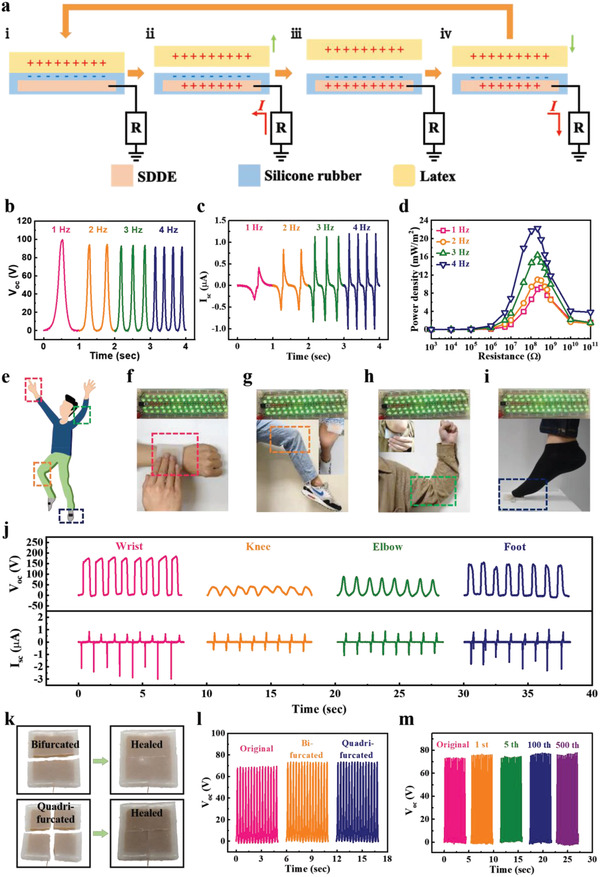
a) Schematic illustration of the working principle for the SDDE‐TENG. The electrical output b) *V*_oc_ and c) *I*_sc_ under various working frequencies ranging from 1 to 4 Hz for the SDDE‐TENG. d) Power density with different resistances of external loads. e) Demonstration of use of the SDDE‐TENG on different body parts: f) Wrist, g) knee, h) elbow, and i) foot. Their relative j) *V*_oc_ and *I*_sc_. k) Photographic images of the SDDE‐TENG from cutting to self‐healing. l) *V*_oc_ for the self‐healed SDDE‐TENG following bifurcation and quadrifurcation at 25 °C. m) *V*_oc_ for the SDDE‐TENG over 500 sequential bifurcating healing cycles at 25 °C. Note that the profiles were recorded at 4 Hz with a contact force of 1 N for the various healing process over a healing time of 10 min.

Owing to its outstanding self‐healing efficiency and flexibility, the prepared SDDE has considerable potential as a soft material for use in next‐generation soft devices, wearable electronics, and bionic electronic skins. We provide a proof‐of‐concept validation of the use of this SDDE as a flexible energy device by fabricating a single‐electrode TENG where the SDDE acts as the conducting electrode (**Figure** **4**a).^[^ ^55^, ^56^, ^57^ ^]^ The relative electrochemical properties of the SDDE were shown in Figures S10–S12 in the Supporting Infromation. Due to the synergy between triboelectrification and electrostatic induction, the SDDE‐TENG successfully generated a continuous electric output by successive contact and separation with the skin and the SDDE‐TENG.^[^ ^55^, ^56^, ^57^ ^]^ Figure S13 in the Supporting Information displays the electric simulation profiles depicting the triboelectricity generation process of the SDDE‐TENG at an open‐circuit state. In addition, Figure 4b,c, respectively presented the output open‐circuit voltage (*V*_oc_) and the short‐circuit current (*I*_sc_) of the SDDE‐TENG with a contact force 2 N at different contact/separation frequencies (1–4 Hz), while the corresponding plots for the relative transferred charge (*Q*_tr_) was given in Figure S14 in the Supporting Information. The continuously stable signals for all three outputs (*V*_oc_, *Q*_tr_, and *I*_sc_) generated by the SDDE‐TENG indicate that SDDE can be successfully employed as a single electrode in the TENG. To determine the outputs of the SDDE‐TENG, the voltage, current density and power density were explored via connecting different external loads, respectively, (1–100 GΩ) and tested at different frequencies from 1 to 4 Hz (Figure 4d and Figures S15 and S16, Supporting Information). As observed, the voltage enhanced rapidly as the load resistance was above 1 MΩ. In addition, the current density remained constant as the load resistance was less than 1 MΩ. The power was calculated by the formula *P* = *IV* (where *P*, *I*, and *V* are the output power, current density, and voltage). As frequencies increased from 1 to 4 Hz, the relative maximum outputs increased from ≈9.22 to ≈22.25 mW m^−2^ and the relative resistances located in the range of 220–330 MΩ. In addition, as shown in Figure S17 in the Supporting Information, the SDDE‐TENG exhibited a good durability after 20 000 contact/separation cycles. Figure S18 in the Supporting Information showed the output voltage for different contact materials. For a wearable electronic demonstration, the SDDE‐TENG was fitted on different parts of the human body, including the wrist, knee, elbow, and foot, to act as a mechanical energy harvester (Figure 4e–i). Figure 4j and Figure S19, Supporting Information summarize the *V*_oc_, *I*_sc_, and *Q*_tr_ values recorded for the different parts of the body, with *V*_oc_ values of 175, 35, 80, and 145 V being recorded for the wrist, knees, elbow, and foot, respectively. Considering the practical use, TENGs may suffer from different mechanical impacts. As a result, developing TENGS with self‐healing properties is desired. To investigate the self‐healing ability of the SDDE‐TENG, the electrical performance of the SDDE‐TENG was examined using bifurcating and quadrifurcating healing procedures at ambient temperature (Figure 4k). In both cases, the self‐healing time was 10 min, and the outputs from the pristine and healed SDDE‐TENGs were comparable (Figure 4l and Figure S20, Supporting Information). The SDDE‐TENG was then subjected to cyclic bifurcated cutting. More specifically, after 500 sequential bifurcating self‐healing cycles, the *V*_oc_, *Q*_ir_, and *I*_sc_ values of the SDDE‐TENG displayed an identical intensity to that of the pristine SDDE‐TENG (Figure 4m and Figure S21, Supporting Information), indicating the excellent recovery of the SDDE‐TENG.

**Figure 5:**
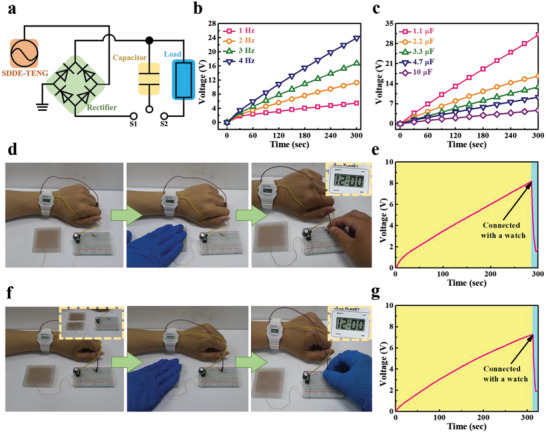
a) Equivalent circuit integrated with the SDDE‐TENG as a mechanical harvesting system. b) Time dependence of the accumulated voltage for charging a 1.1 µF commercial capacitor with different working frequencies using the SDDE‐TENG. c) Time dependence of the accumulated voltage for charging different commercial capacitors using the SDDE‐TENG at a working frequency of 4 Hz. d) Photographic images of the harvesting of energy using the SDDE‐TENG to drive an electronic watch, and e) its real‐time charge/discharge curve. f) Photographic images of the harvesting of energy using the self‐healed SDDE‐TENG to drive an electronic watch, and g) its real‐time charge/discharge curves. The healing time was 10 min.

The SDDE‐TENG was also integrated with capacitors to power commercial electronic devices. The equivalent circuit model encompassing the SDDE‐TENG, a rectifier, and a capacitor is illustrated in **Figure** **5**a, where tests were performed using a 5 × 5 cm^2^ SDDE‐TENG with a contact force of 2 N at 4 Hz. The charging profiles for the 1.1 µF capacitor at different working frequencies are shown in Figure 5b, and the charging profiles for the different capacitors charged by the SDDE‐TENG at 4 Hz are shown in Figure 5c. As indicated, the charging voltage reached up to 31 V within 300 s for a 1.1 µF capacitor, while a 10 µF capacitor was charged to 4.7 V within the same time period of 300 s. As demonstrated in Figure 5d–e and Figure S22 in the Supporting Information an electronic watch and an electronic calculator were powered when the charged capacitor began to discharge. The real‐time charge/discharge curves for the electronic watch and the electronic calculator are shown in Figure 5e and Figure S22 in the Supporting Information, respectively. In addition, Movie S1 in the Supporting Information displays demonstrations of driving the devices using harvested biomechanical energy. Accordingly, the healed SDDE‐TENG (10 min healing time) from bifurcating retained 90% of its function of triboelectric generation with an identical charging time of 300 s (Figure 5f,g and Movie S2 in the Supporting Information).

**Figure 6:**
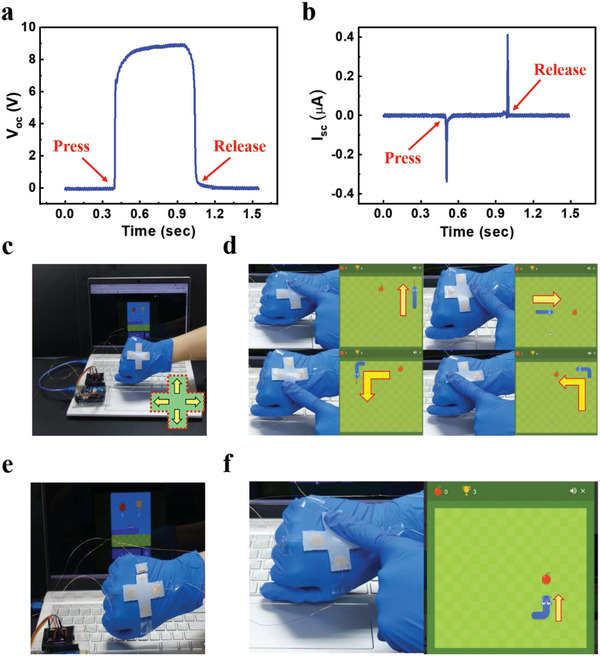
Multifunctional SDDE‐TENG as wearable self‐powered human–machine interfaces. a) *V*_oc_ and b) *I*_sc_ during finger touching and releasing the SDDE‐TENG. c,d) Demonstration of the classical computer game “Snake” using the SDDE‐TENG. e,f) Demonstration of the classical computer game “Snake” with the self‐healed SDDE‐TENG. The healing time was 10 min.

The SDDE‐TENG can not only serve as a power generator but also be able to act as self‐powered human–device interfaces by integration with microcontrollers. The real‐time signals generated by touch are shown in **Figure** **6**a,b. Figure 6c presents the SDDE‐TENG‐based self‐powered controller with four arrow keys. Four elements were used as the “up,” “left,” “right,” and “down” keys, respectively. By the combination with the microcontroller, it can be performed to play a computer game “Snake Game” (Figure 6d and Movie S3 in the Supporting Information). More specifically, the controller split by the scissors was able to heal itself under ambient environment within 10 min and displayed an identical performance to the pristine one (Figure 6e,f and Movie S4 in the Supporting Information).

**Figure 7:**
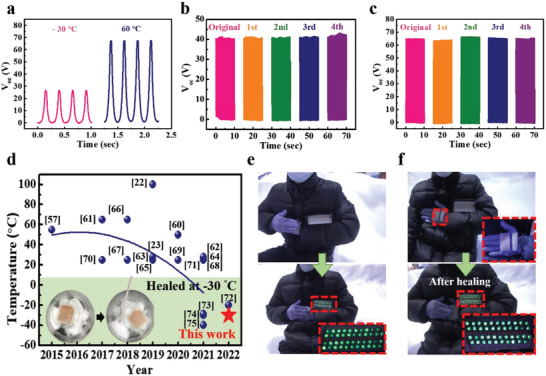
a) *V*_oc_ for the SDDE‐TENG at 60 and −30 °C. b) *V*_oc_ for the self‐healed SDDE‐TENG at −30 °C (healing time = 12 h) and c) *V*_oc_ for the self‐healed SDDE‐TENG at 60 °C (healing time = 1 h). d) Progression toward a self‐healable TENG. The general trend showed an improvement in the self‐healing ability toward harsh environments. Our work (red star) represents the first report of self‐healing at an extremely low subzero temperature.^[^ ^22^, ^23^, ^57^, ^60^, ^61^, ^62^, ^63^, ^64^, ^65^, ^66^, ^67^, ^68^, ^69^, ^70^, ^71^, ^72^, ^73^, ^74^, ^75^ ^]^ e,f) Demonstration of using the SDDE‐TENG and the self‐healed SDDE‐TENG to illuminate 48 LEDs in a snowfield.

With the aim of developing a universally healable material that undergoes self‐repairing under harsh conditions, we investigated the stability and the recovery efficiency of our SDDE‐TENG in two virtual extreme environments, i.e., *T* = 60 °C, RH = 0–5%, (Sahara Desert‐like) and *T* = −30 °C, RH = 80–90% (Siberia‐like). More specifically, for the *T* = 60 °C and RH = 2% conditions, the *V*_oc_ of the undamaged SDDE‐TENG was similar to that recorded at ambient temperature (i.e., 70 V) (**Figure** **7**a). However, the *V*_oc_ at *T* = −30 °C and RH = 80% was only 40 V, which is lower than that obtained at ambient temperature. Accordingly, the *I*_sc_ at *T* = −30 °C and RH = 80% also showed a decreased value (Figure S23, Supporting Information). The performances of the SDDE‐TENG under different temperatures or different relative humidity were shown in Figures S24 and S25 in the Supporting Information, respectively.^[^ ^58^, ^59^ ^]^ This result was associated with the high humidity, which severely reduced the generation of triboelectricity.^[^ ^59^ ^]^ To evaluate the recovery efficiency of the SDDE‐TENG under these two extreme conditions, the SDDE‐TENG was subjected to a successive bifurcating/healing procedure at *T* = −30 °C and RH = 80% (with a 12 h healing time), and at *T* = 60 °C and RH = 2% (with a 1 h healing time); the relative *V*_oc_, *Q*_tr_, and *I*_sc_ values are shown in Figure 7b,c and Figure S26 in the Supporting Information, respectively. It is worth noting that under both simulated environments, the behavior of the SDDE‐TENG was almost identical to that of the pristine SDDE‐TENG, even after four bifurcating/healing cycles.

Finally, we compared our results with those of other self‐healable TENGs reported since 2012 in terms of their self‐healing abilities versus temperature (Figure 7d).^[^ ^22^, ^23^, ^57^, ^60^, ^61^, ^62^, ^63^, ^64^, ^65^, ^66^, ^67^, ^68^, ^69^, ^70^, ^71^, ^72^, ^73^, ^74^, ^75^ ^]^ In addition, we also compared with previous works on antifreezing TENGs in Table S3 in the Supporting Information.^[^ ^29^, ^30^, ^31^, ^72^, ^73^, ^74^, ^75^, ^76^, ^77^, ^78^, ^79^, ^80^ ^]^ Upon comparison of these results, it appears that our material is the multifunctional TENG that exhibits a self‐healing ability at such low temperatures. As an extension to demonstrate its activity after exposure to low temperatures, Figure 7e,f and Movies S5 and S6 in the Supporting Information show that our SDDE‐TENG can light 48 parallel LEDs both before and after self‐healing in a snowfield. This excellent self‐healing recovery under harsh conditions, in addition to the retained elasticity, flexibility, and antiaging properties, therefore indicate the potential application of our SDDE‐TENG in soft electronics for displays, bionic skins, and wearable sensors aimed at monitoring motion and physiological well‐being.

## 3 Conclusions

In summary, we developed an environmentally inert water‐based elastomer with extremely efficient self‐healing properties for TENG applications. To the best of our knowledge, the self‐healing efficiency of 96% achieved within 2 h at room temperature is the highest value reported to date. In addition, the key to the obtained superior elasticity and excellent self‐healing efficiency under extreme conditions (i.e., temperature (*T*) = 60 °C, RH = 0–5%, and *T* = −30 °C, RH = 80–90%) was the combination of a symmetric aromatic structure with double‐terminal disulfides and a multiple hydrogen‐bonding crosslinked network. Furthermore, the SDDE‐TENG (SDDE symmetric double‐terminal aromatic disulfide elastomer) was found to exhibit reliable electrical outputs, which could be adapted to many parts of the human body, and importantly, it showed repeatable self‐healing properties over the temperature range of −30 to 60 °C. Our results are therefore expected to facilitate the application of such materials in wearable energy and electronics applications in wide environmental conditions.

## 4 Experimental Section

### Materials and Chemicals

4‐Mercaptobenzoic acid (technical grade 90%, C_7_H_6_O_2_S, chemical abstracts service (CAS) No. 1074368), iodine plates (CAS No. 7553562), ethanol (C_2_H_5_OH, CAS No. 64175), trimethylamine (C_3_H_9_N, CAS No. 75‐50‐3), sodium thiosulfate (Na_2_S_2_O_3_, CAS No. 7772987), *N,N*‐dimethylacetamide (C_4_H_9_NO, CAS No. 127195), l‐cystine (C_6_H_12_N_2_O_4_S_2_, CAS No. 56893), methacrylic anhydride (C_8_H_10_O_3_, CAS No. 760930), thionyl chloride (SOCl_2_, CAS No, 7719097), and [2‐(methacryloyloxy)ethyl]dimethyl‐(3‐sulfopropyl)ammonium hydroxide (SBMA, CAS No. 3637261) were purchased from Sigma‐Aldrich (St. Louis, MO, USA). Hydrochloric acid (HCl; CAS No. 7637010) and sodium hydroxide (NaOH; CAS No. 1310732) were purchased from Riedel‐de Haën (Germany). Ammonium persulfate ((NH_4_)_2_S_2_O_8_) was purchased from J.T. Baker (New Jersey, USA).

### Synthesis of the SDDE

Initially, 4‐mercaptobenzoic acid (0.31 g) and iodine plates (0.26 g) were dissolved together in ethanol (5 mL) under stirring until completely dissolved, and trimethylamine (0.75 mL) was then added to the above solution under stirring. After continuing stirring for 16 h, a 10 wt% sodium thiosulfate solution was added to quench any excess iodine. Hydrochloric acid (10 mm; 10 mL) was then added to the muddy solution to form a precipitate, which was filtered and dried under vacuum. The dried crude product was recrystallized by immersion in a mixture of *N,N*‐dimethylacetamide and deionized water (1:1) to obtain 4,4‐dithiobisbenzoic acid. Separately, cystine (3 mmol) was dissolved in an aqueous sodium hydroxide solution (9 mL, 1 m) at 60 °C with stirring and then methacrylic anhydride (3 mL) was added at a rate of 0.5 mL min^−1^ under stirring for 6 h. The obtained 4,4‐dithiobisbenzoic acid (6 mmol) was dissolved in thionyl chloride and refluxed at 70 °C for 2 h. After cooling to room temperature, the above solution was added to the cystine/methacrylic anhydride solution (9 mL) and incubated at 60 °C for 8 h under gentle stirring to obtain a symmetric aromatic double‐terminal disulfide polymer (DS). Finally, zwitterion branches were grafted onto the polymer by the addition of [2‐(methacryloyloxy)ethyl]dimethyl‐(3‐sulfopropyl)ammonium hydroxide (60 mmol) to the as‐synthesized polymer solution (9 mL). Finally, radical polymerization was conducted under gentle stirring at 65 °C for 72 h in the presence of ammonium persulfate (0.1 g) to obtain the final product, denoted as the SDDE.

### Characterization

FTIR (PerkinElmer Spectrum 100, Waltham, Massachusetts, USA) was used to identify the functional groups of the cystine/methacrylic anhydride solution and the SDDE. The FTIR spectra were recorded between 400 and 4000 cm^−1^ with a resolution of 4 cm^−1^ over 32 scans. XPS (Thermo Fisher Scientific, ESCALAB Xi^+^, UK) was conducted to investigate the bonding present in the SDDE and to carry out high‐resolution elemental analysis. A microforce testing system (Tytron 250, Eden Prairie, Minnesota, USA) was used to perform the standard tensile strain–stress tests and self‐healing cyclic tensile tests at a stretching rate of 0.1 mm s^−1^. The healing time for every self‐healing cyclic tensile experiment is 2 h. The tensile stress–strain test was also performed with different loading speed, including 10, 30, and 50 mm min^−1^. Each sample used for the microforce tensile test was cut into a rectangular shape (5 mm × 2 mm × 0.3 mm). A LT‐DSC (Netzsch, Germany) was used to analyze the thermal profile and estimate the glass transition temperature (*T*_g_) of the SDDE. The temperature range of the LT‐DSC was from −75 to 60 °C with heating at a rate of 5 °C min^−1^. A Discovery Hybrid Rheometer (HR‐1, Newcastle, USA) was used to evaluate the storage modulus, the loss modulus, and the complex viscosity of the SDDE at an angular frequency of 0.1–100 rad s^−1^ with 5% strain, and at a strain of 0.005–1000% at 1 rad s^−1^ and ambient temperature. To evaluate the aging of the SDDE, the SDDE film was placed under UV radiation (0.07 W cm^−1^) under ambient conditions for 4 weeks, after which time the microforce tensile test and the bifurcated self‐healing test were repeated.

### Fabrication and Electrical Characterization of the SDDE‐TENG

The SDDE‐TENG was prepared by encapsulating SDDE between a silicone rubber mold (50 mm × 50 mm × 1 mm) with a 40 mm × 40 mm × 1 mm trough and a silicone rubber cover (50 mm × 50 mm × 1 mm), where the SDDE was placed in the trough working as the conducting layer and the silicone rubber cover acted as the triboelectrically charged layer. To ensure good contact between the materials, the TENG device was stored at 50 °C for 1 h until all materials were cured. The *V*_oc_ and *Q*_tr_ values, in addition to the charge/discharge curves, were measured using a Keithley 6514 electrometer, while *I*_sc_ was measured using a low‐noise current amplifier (SR570, Stanford Research System).

### Practical Demonstration of the SDDE‐TENG

To evaluate the practical application of the SDDE‐TENG system, it was integrated with a capacitor to power up the external loads, and switches were used to control the working states between the charge and discharge operations. The collected energy was used to drive an electronic watch and a calculator. The SDDE‐TNEG was further evaluated as a self‐powered controller containing four SDDE electrodes (10 mm × 10 mm) embedded in silicone rubber. By the combination with the microcontroller, it was performed to play the classical computer game “Snake Game.” To evaluate the self‐healing ability of the TENG, all SDDE‐TENGs were cut in a bifurcated or quadrifurcated manner using a razor and then healed under ambient conditions. The *V*_oc_ and *Q*_tr_ values, along with the charge/discharge curves, were measured using a Keithley 6514 electrometer, while *I*_sc_ was measured using a low‐noise current amplifier (SR570, Stanford Research System).

### Evaluation of the SDDE‐TENG in Extreme Environments

To further testing of the self‐healing ability of the prepared SDDE‐TENG system in extreme environments, its self‐healing properties were evaluated under various harsh conditions, including the freezing state (*T* = −30 °C, RH = 80%) and the broiling state (*T* = 60 °C, RH = 2%). To detect the outputs with the different conditions, the SDDE‐TENG was placed in a vacuum oven (*T* = 60 °C and RH = 2%) and a container (*T* = −30 °C and RH = 80%), respectively. Note that the container was full of dry‐ice, ethylene glycol, and ethanol, achieving the subzero temperature.^[^ ^81^ ^]^ The above tests were immediately carried out when the SDDE‐TENG were moved away from the environments. All SDDE‐TENGs were cut by a razor, followed by slicing of the cracked surface, and the film was healed for 12 and 1 h in the freezing and broiling states, respectively.

## Conflict of Interest

The authors declare no conflict of interest.

## Supporting information

Supporting Information

Supplemental Movie 1

Supplemental Movie 2

Supplemental Movie 3

Supplemental Movie 4

Supplemental Movie 5

Supplemental Movie 6

## Data Availability

The data that support the findings of this study are available from the corresponding author upon reasonable request.
