# Supplementary material for: An Environmental‐Inert and Highly Self‐Healable Elastomer Obtained via Double‐Terminal Aromatic Disulfide Design and Zwitterionic Crosslinked Network for Use as a Triboelectric Nanogenerator
Source: Adv Sci (Weinh). 2022 Dec 1;10(2):2202815. doi: 10.1002/advs.202202815 (PMC9839881; doi:10.1002/advs.202202815)
Supplement: Supplementary file 1 — Supporting Information [file ADVS-10-2202815-s005.pdf]

ADVANCED SCIENCE
Open Access

Supporting Information

for *Adv. Sci.*, DOI 10.1002/advs.202202815

An Environmental-Inert and Highly Self-Healable Elastomer Obtained via Double-Terminal Aromatic Disulfide Design and Zwitterionic Crosslinked Network for Use as a Triboelectric Nanogenerator

Syun-Hong Chou, Hong-Wei Lu, Ta-Chung Liu, Yi-Ting Chen, Yen-Lin Fu, Yung-Hsin Shieh, Ying-Chih Lai and San-Yuan Chen**

Supporting Information

An Environmental-inert and Highly Self-healable Elastomer Obtained via Double-terminal Aromatic Disulfide Design and Zwitterionic Crosslinked Network for Use as a Triboelectric Nanogenerator

Syun-Hong Chou, Hong-Wei Lu, Ta-Chung Liu, Yi-Ting Chen, Yen-Lin Fu, Yung-Hsin Shieh, Ying-Chih Lai,and San-Yuan Chen**

S. H. Chou, Y. L. Fu, Prof. S. Y. Chen
Department of Materials Science and Engineering,
National Yang Ming Chiao Tung University, Hsinchu 30010, Taiwan
Email: sanyuanchen@nycu.edu.tw

H. W. Lu, Y. T. Chen, Prof. Y.C. Lai
Department of Materials Science and Engineering,
National Chung Hsing University, Taichung 402, Taiwan
Email: yclai@nchu.edu.tw

Prof. T. C. Liu
Department of Biomedical Engineering,
National Yang Ming Chiao Tung University, Taipei 112, Taiwan

Y. H. Shieh
Department of Materials Science and Engineering,
National Tsing Hua University, Hsinchu 300044, Taiwan

Prof. Y. C. Lai
Innovation and Development Center of Sustainable Agriculture,
i-Center for Advanced Science and Technology,
National Chung Hsing University,
Taichung 402, Taiwan

Prof. S. Y. Chen
Graduate Institute of Biomedical Science,

China Medical University, Taichung City 406040, Taiwan
Frontier Research Centre on Fundamental and Applied Sciences of Matters,
National Tsing Hua University, Hsinchu 300044, Taiwan
School of Dentistry, College of Dental Medicine,
Kaohsiung Medical University, Kaohsiung City 80708, Taiwan

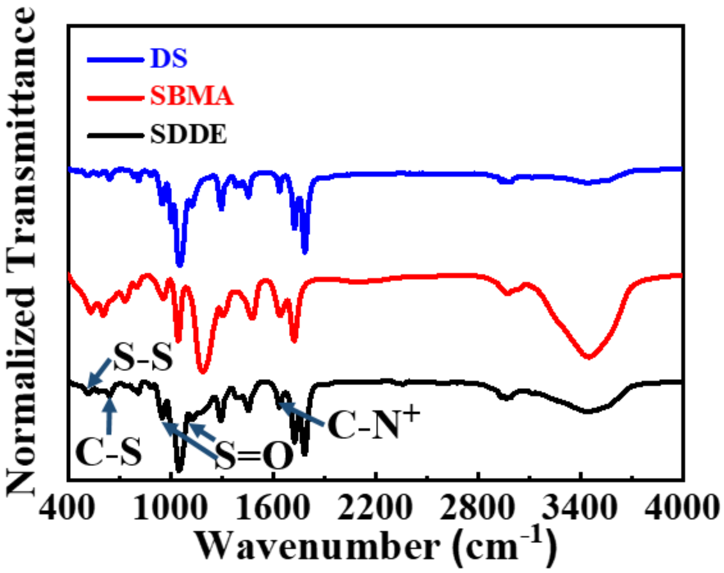

Figure S1. FTIR spectra of the DS, the SBMA and the SDDE. The presence of the SBMA functional group is confirmed by the following peaks: -S=O stretching at 1046 and 1191 cm^{-1}, C-S stretching at 606 cm^{-1}, C-N^+ stretching at 1646 cm^{-1}, S-S stretching at 516 cm^{-1}, and O-C=O stretching at 1727 cm^{-1}.

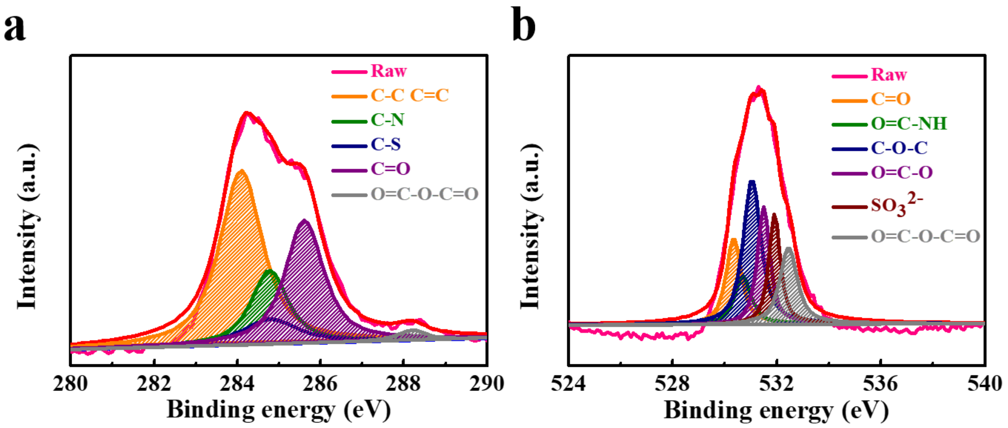

Figure S2. High-resolution of XPS spectrum of a) the C 1s region and b) the O 2p region for the SDDE. The C 1s profile is deconvoluted into several peaks including C-C/C=C, C-N, C-S, and C=O bonding centered at 284.1, 284.8, 284.9, and 285.7 eV, respectively. However, these four peaks were also present in the spectra of the synthetic precursors, and so cannot be used to confirm the synthesis of the SDDE. In contrast, the characteristic O=C-O-C=O anhydride bonding energy at 288.2 eV indicates the presence of a new covalent bond between the master chains and the SBMA side chains, thereby confirming the successful synthesis of the SDDE. The O=C-O-C=O bond was also detected in the O 2p region at 532.5 eV. Finally, the peaks at 530.4, 530.7, 531, 531.5, and 531.9 eV were attributed to C=O, O=C-NH, C-O-C, O=C-O, and SO_3^{2-}, respectively.

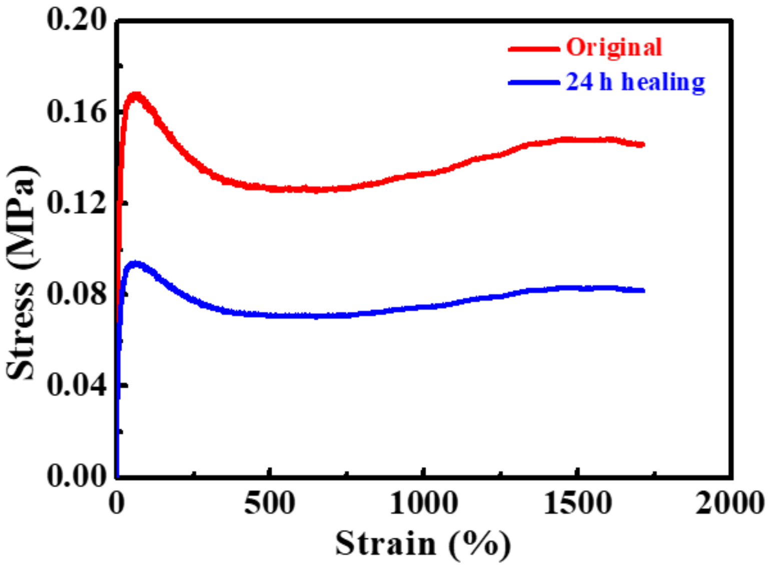

Figure S3. Tensile stress-strain profile of the original and healed zwitterionic polymer.

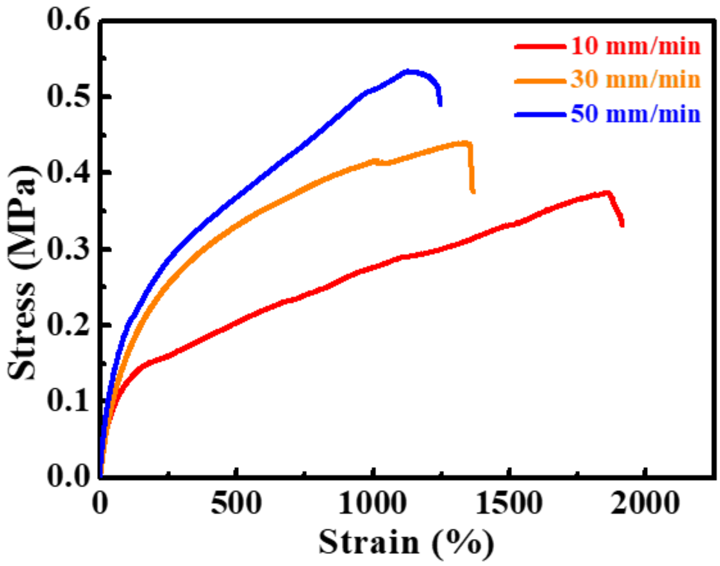

Figure S4. Tensile stress-strain profile of the SDDE with different loading speeds.

Table S1. High-resolution XPS of the surface elemental analysis and the respective deconvoluted S 2p analyses of the SDDE before and after self-healing

	**S 2p**	**C 1s**	**N 1s**	**O 1s**	**Total**
**Before self-healing**	3.4%	63.6%	6.9%	26.1%	100%
**After self-healing**	5.1%	58.1%	13.3%	23.5%	100%
	**SO_3^{2-} $2p_{3/2}$**	**SO_3^{2-} $2p_{1/2}$**	**S-S $2p_{3/2}$**	**S-S $2p_{1/2}$**	**Total**
**Before self-healing**	62.7%	30.2%	4.4%	2.7%	100%
**After self-healing**	52.2%	29.3%	9.7%	8.8%	100%

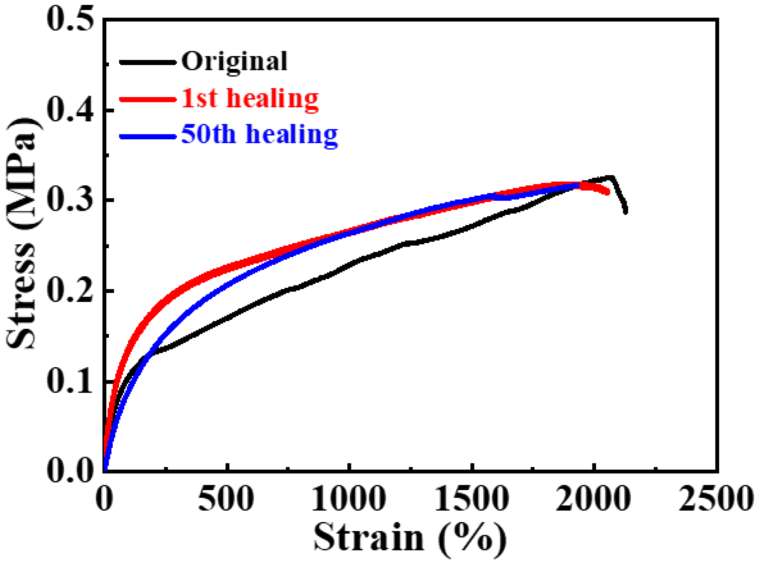

Figure S5. Tensile stress-strain profile of the original and healed SDDE at 60 °C.

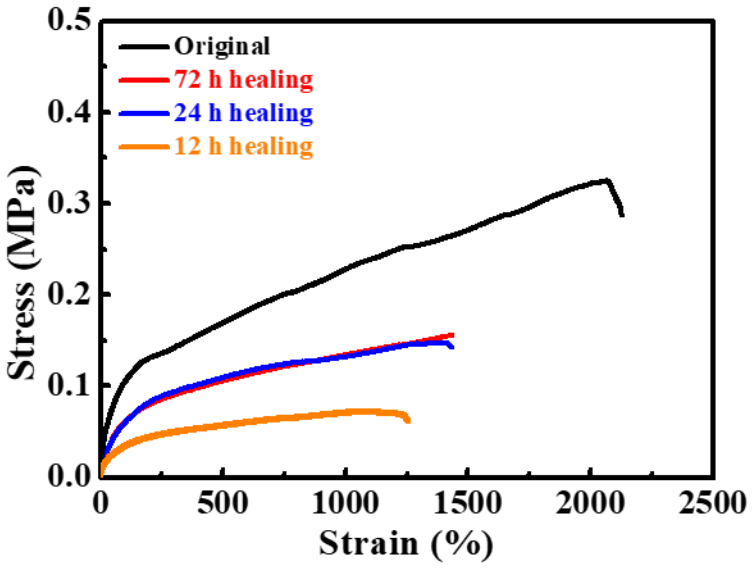

Figure S6. Tensile stress-strain profile of the SDDE at -30 °C with different healing times.

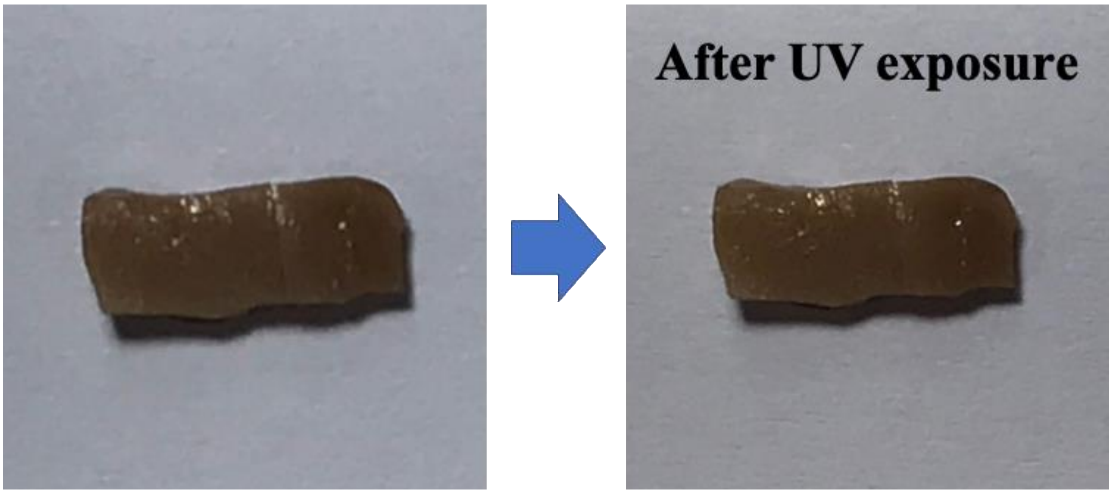

Figure S7. Photographic images of the SDDE before (left) and after (right) 4 weeks of UV light exposure. No material yellowing was observed following exposure.

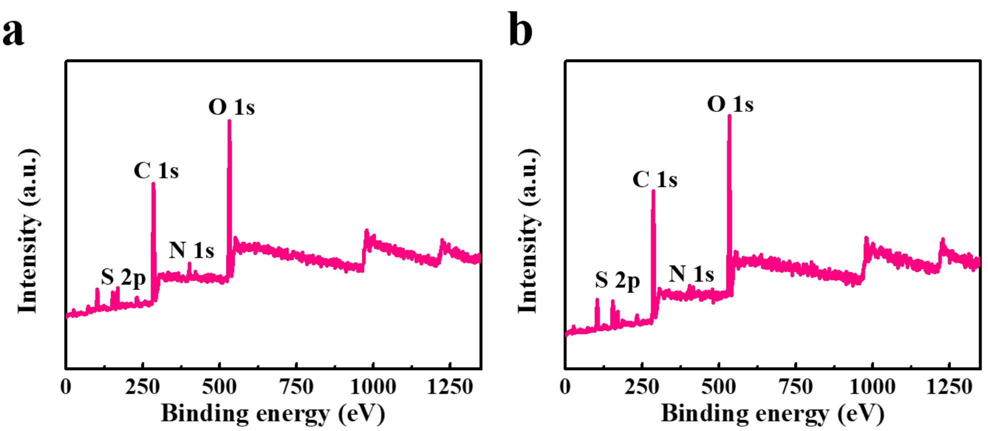

Figure S8. a) XPS full scan survey spectrum of the pristine SDDE. b) XPS full scan survey spectrum of the SDDE after 4 weeks of UV light exposure.

Table S2. XPS surface elemental analysis of the SDDE before and after 4 weeks of UV exposure

	S 2p	C 1s	N 1s	O 1s	Total
**Before UV light exposure**	5.2%	60.2%	5.9%	28.7%	100%
**After UV light exposure**	4.7%	61.1%	5.8%	28.4%	100%

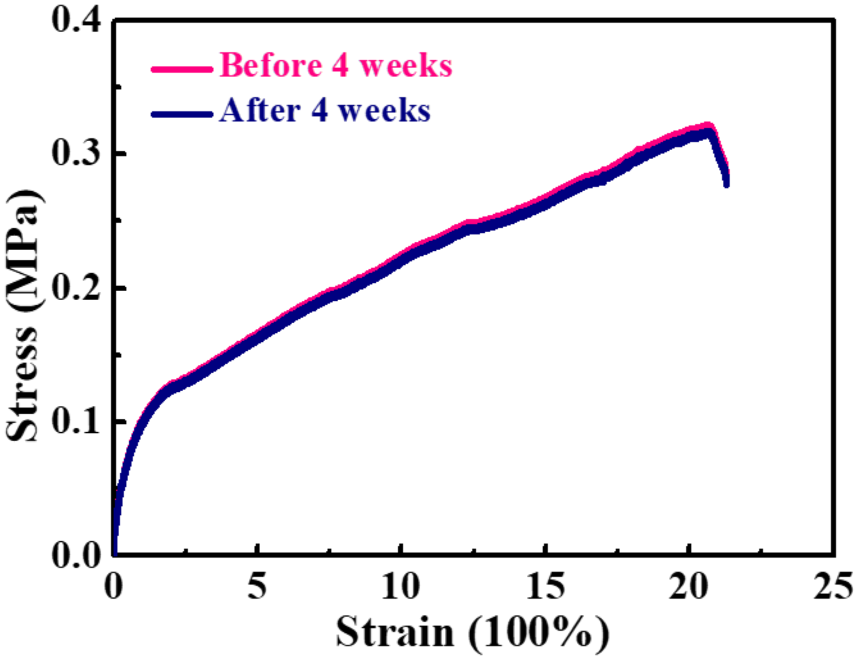

Figure S9. Tensile stress–strain profile of the SDDE before and after 4 weeks of UV light exposure.

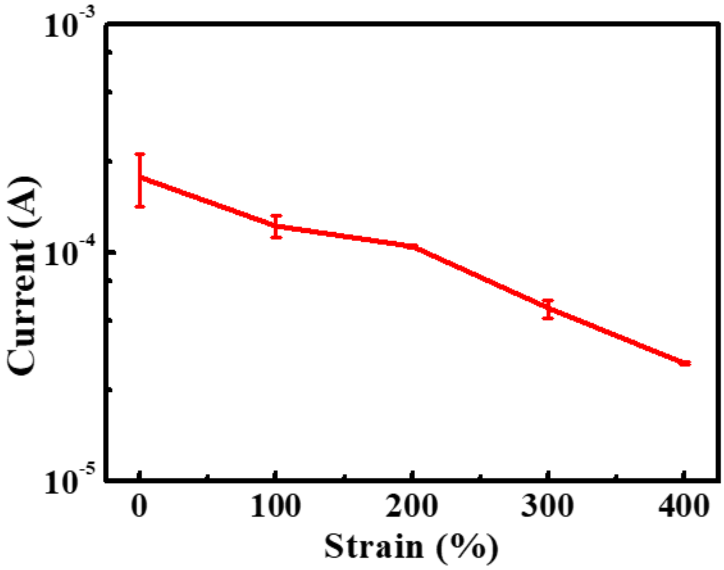

Figure S10. The current of SDDE at different strains.

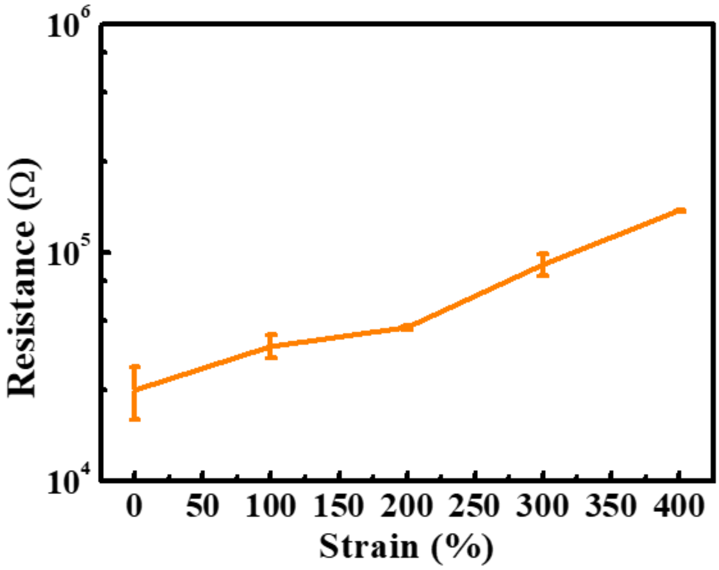

Figure S11. The resistance of SDDE at different strains.

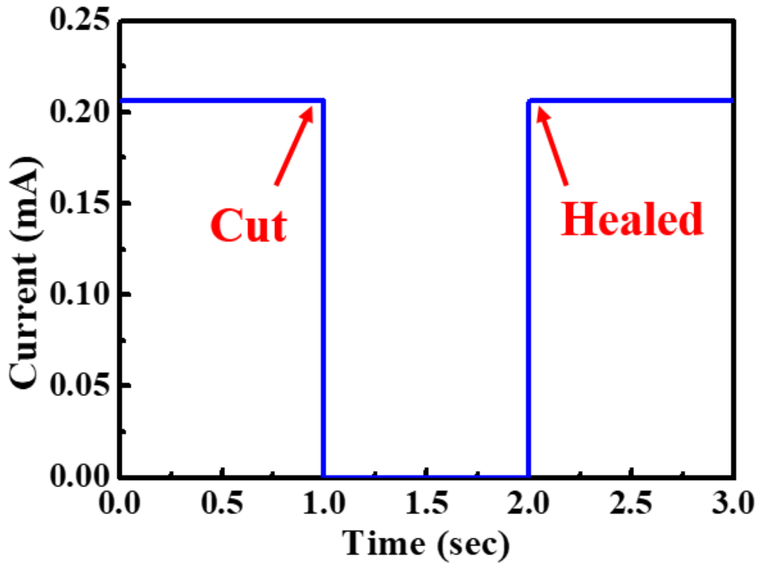

Figure S12. The recovery of current after the cut-and-healed procedure.

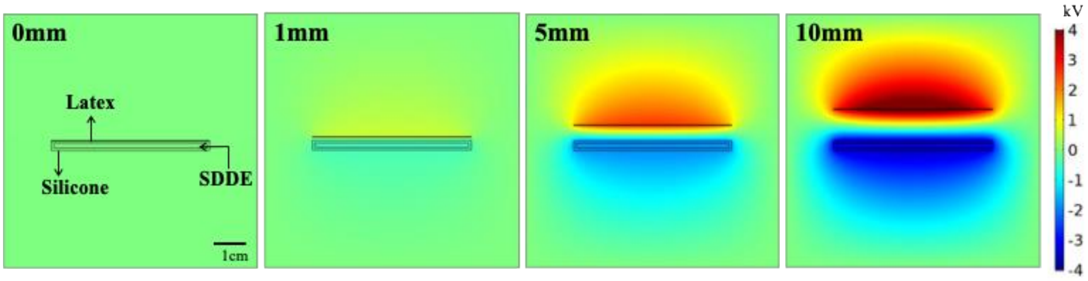

Figure S13. Stimulated electric field distribution under different contact conditions of the open-circuit for the SDDE-TENG.

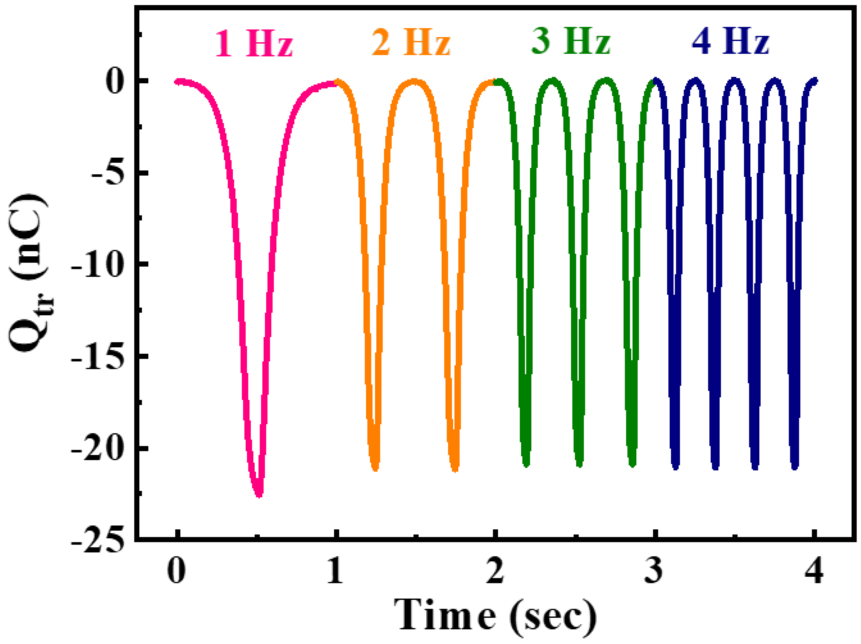

Figure S14. The electrical outputs of Q_{tr} under various working frequencies ranging from 1 to 4 Hz for the SDDE-TENG.

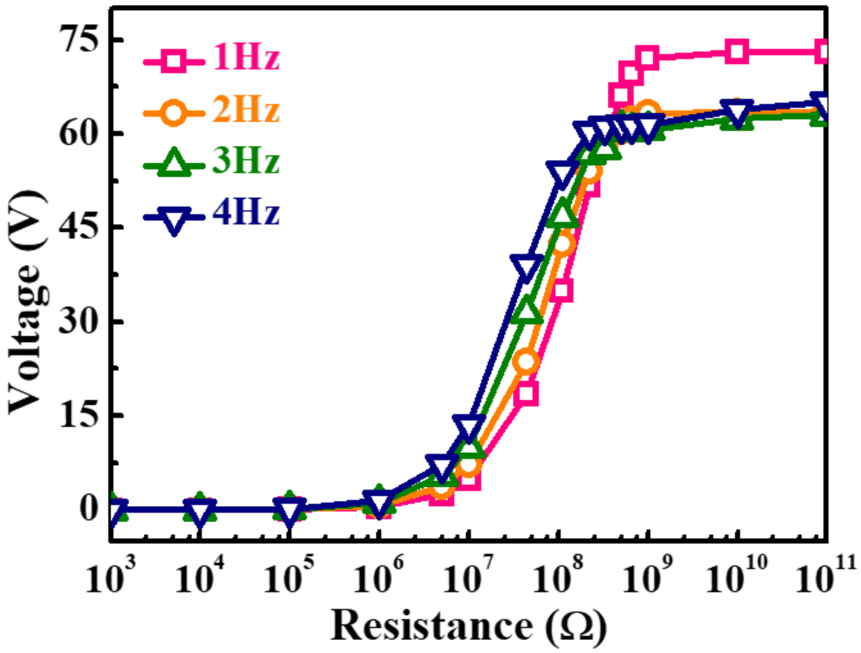

Figure S15. Dependence of the output voltage on difference resistances of external loads.

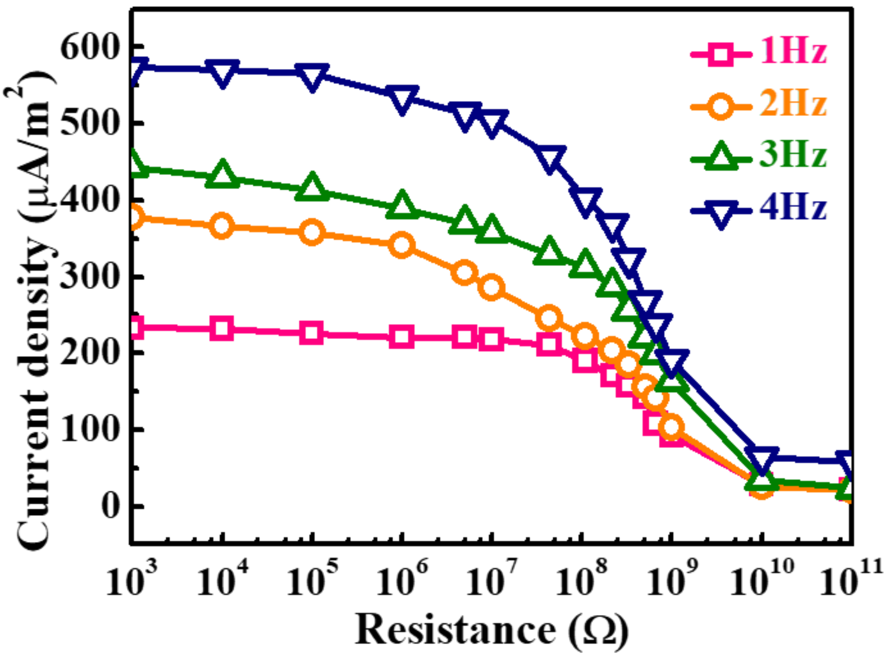

Figure S16. Dependence of the output current density on difference resistances of external loads.

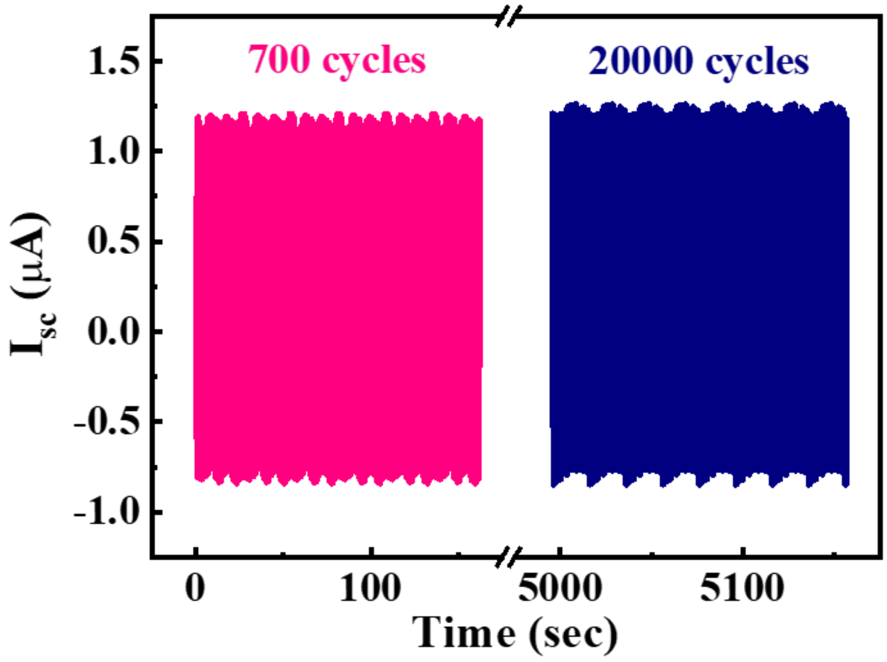

Figure S17. Long-term stability of the performance of I_{sc} after 20,000 cycles at a working frequency of 4 Hz for the SDDE-TENG.

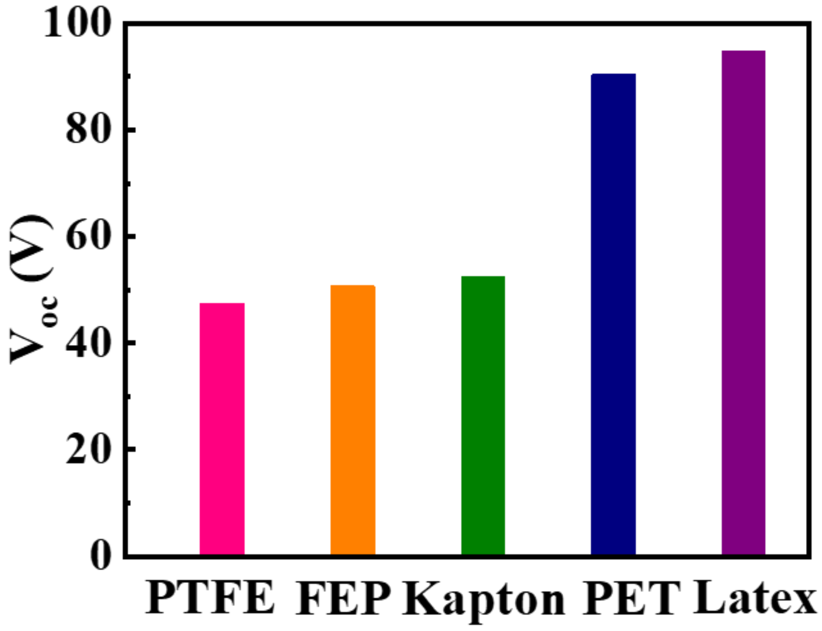

Figure S18. Dependence of open voltage on difference contact materials.

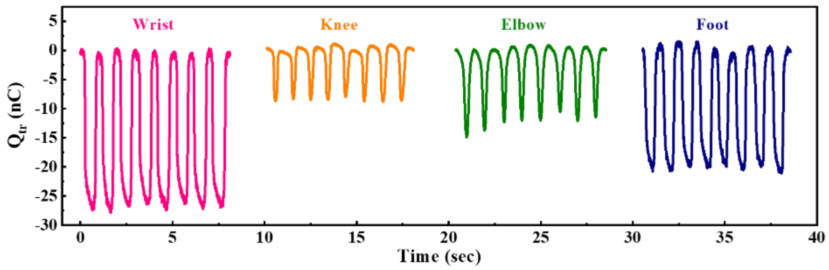

Figure S19. The electrical outputs of Q_{tr} for the SDDE-TENG on different body parts at a working frequency of 4 Hz.

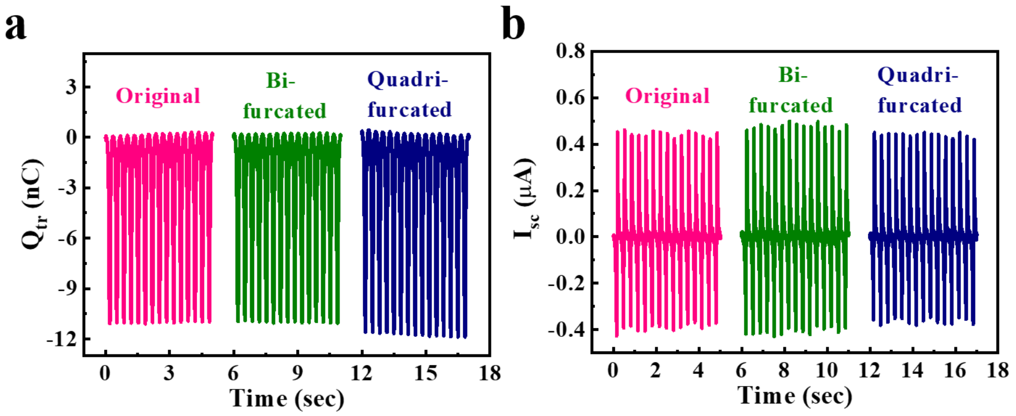

Figure S20. The electrical outputs of a) Q_{tr} and b) I_{sc} of the healed SDDE-TENG following bifurcated and quadrifurcated damage at 25 °C. Note that the profiles were recorded at 4 Hz and the healing time was 10 min.

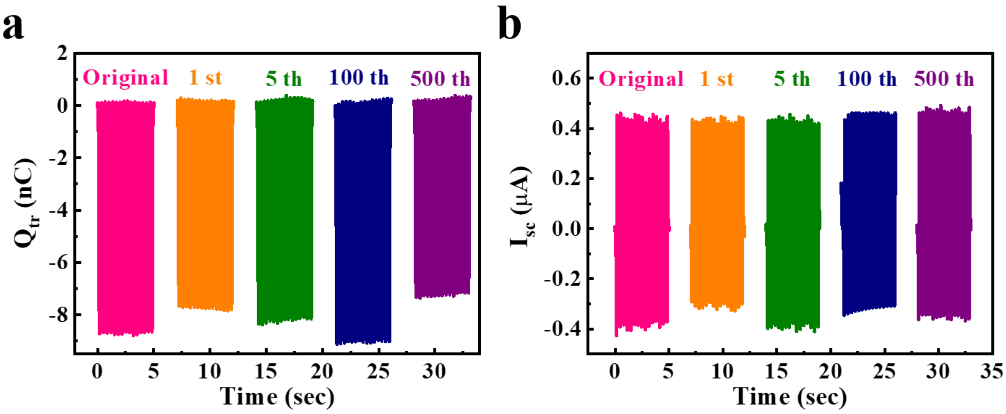

Figure S21. The electrical outputs of a) Q_{tr} and b) I_{sc} for the SDDE-TENG over 500 sequential healing cycles at 25 °C. Note that the profiles were recorded at 4 Hz and the healing time was 10 min.

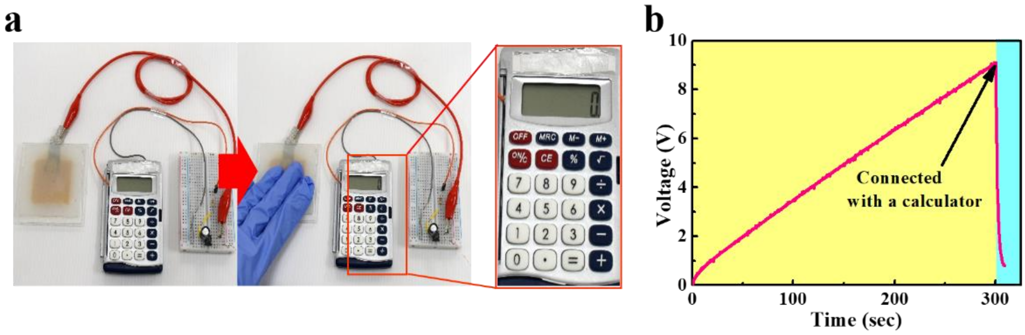

Figure S22. a) Demonstration of charging an electronic calculator by the SDDE-TENG and b) the corresponding charge/discharge curve.

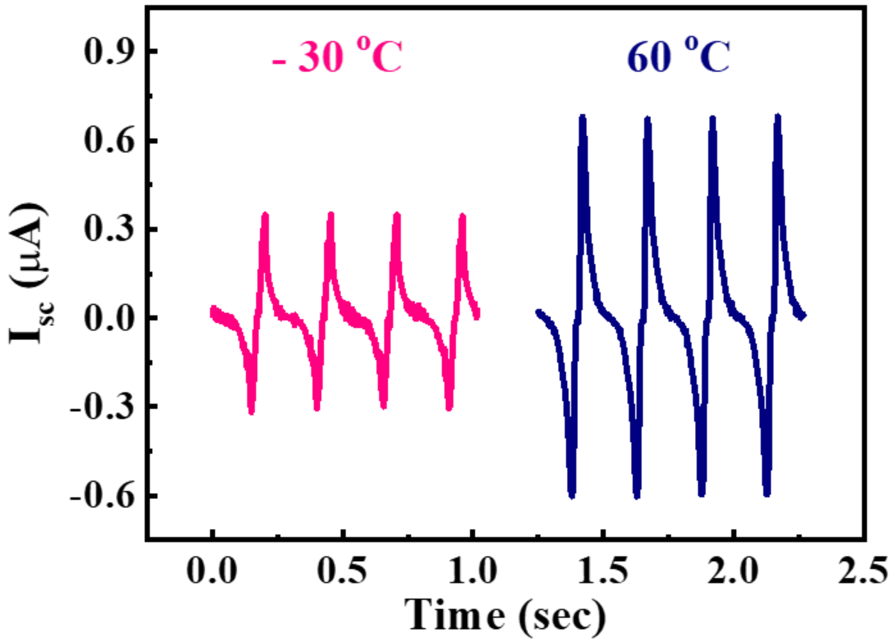

Figure S23. I_{sc} values of the SDDE-TENG at 60 and −30 °C.

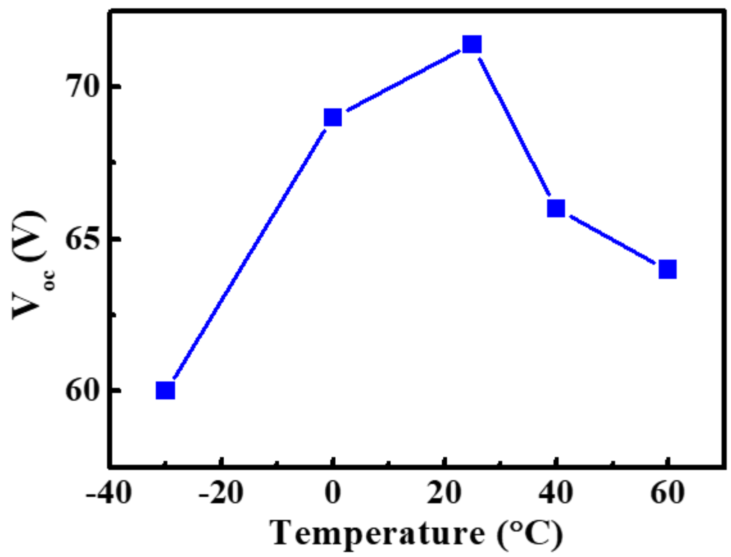

Figure S24. The relationship between the increasing temperature and voltage. All results are performed at the 45~50% humidity.

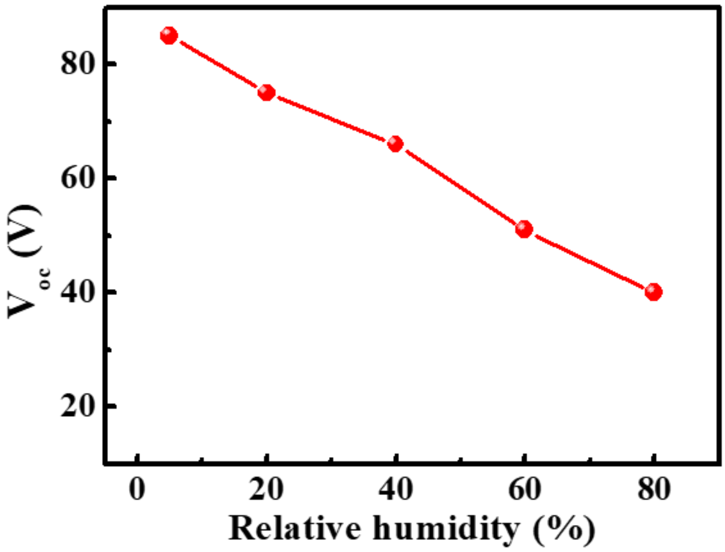

Figure S25. The relationship between the voltage and relative humidity. All results are performed at ambient temperature.

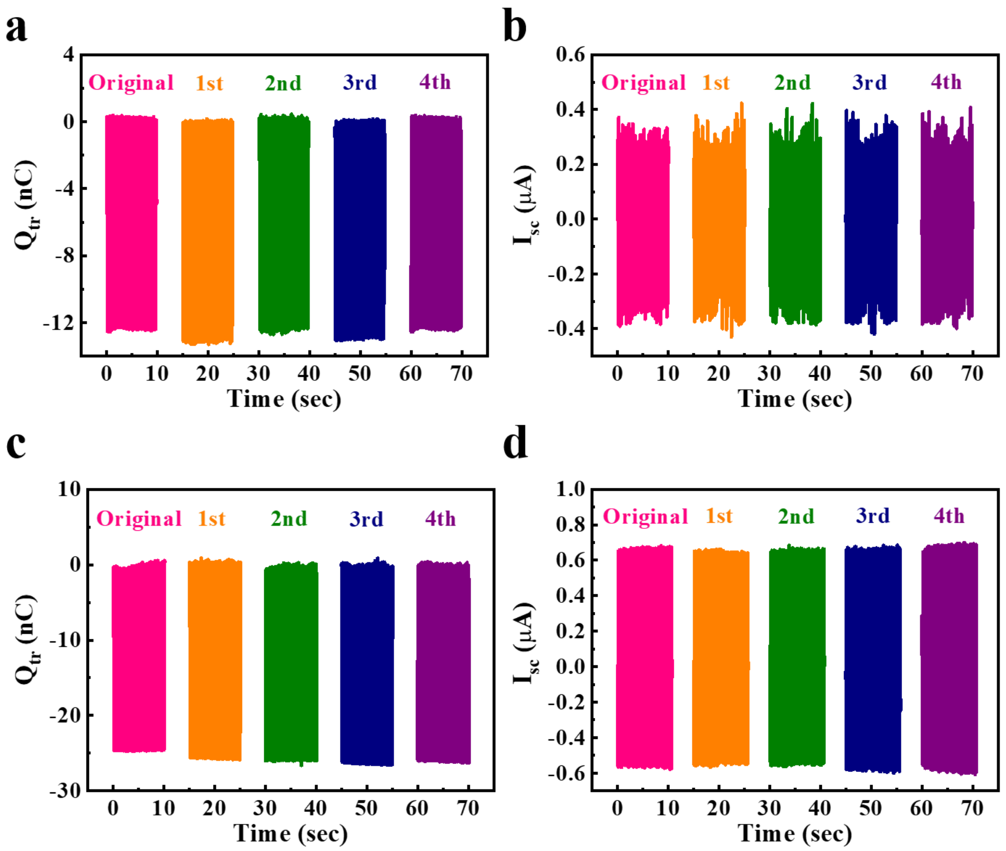

Figure S26. The electrical outputs of a) Q_{tr} and b) I_{sc} for the SDDE-TENG over 4 sequential healing cycles at −30 °C. Note that the profiles were recorded at 4 Hz and the healing time was 12 h. The electrical outputs of c) Q_{tr} and d) I_{sc} for the SDDE-TENG over 4 sequential healing cycles at 60 °C. Note that the profiles were recorded at 4 Hz and the healing time was 1 h.

REF No	Materials of TENG (Triboelectric layer/ electrode)	Outputs from TENG	Anti-freezing	Self-healing	Stretchability	Anti-aging
This work	Silicone rubber/ 4-Mercaptobenzoic acid and [2-(methacryloyloxy)ethyl]dimethyl-(3-sulfopropy l)ammonium hydroxide	~85V, 1μA (4Hz, 5×5 cm^2)	○ (-30 °C)	○ (96 %, 2 h, rt)	○ (2076 %)	○ (4 weeks)
76	Silicone rubber / acrylamide monomer and hydroxyethyl cellulose hydrogel	285 V, 15.5 μA (2.5 Hz, 3×3 cm^2)	○ (-20 °C)	×	○ (~1500 %)	--
77	VHB / cellulose, poly (ethylene glycol) diglycidyl ether organohydrogel	205 V, 1 μA (1 Hz, 3×3 cm^2)	○ (-24 °C)	×	○ (~250 %)	--
31	Polydimethylsiloxane / poly(4-acryloylmorpholine) and propylene carbonate organogel	44 V (2.8 Hz)	○ (-20 °C)	×	○ (~474 %)	--
30	Polydimethylsiloxane / 1-ethyl-3-methylimidazolium dicyanamide, 3-dimethyl (methacryloyloxyethyl) ammonium propane sulfonate, and acrylic acid ionogel	117 V, 14.3 μA (3 Hz, 2.5×2.5 cm^2)	○ (-20 °C)	×	○ (~407 %)	--
78	Ecoflex / alginate and acrylamide hydrogel	380 V (2 Hz, 3×3 cm^2)	○ (-20 °C)	×	×	--
79	Ecoflex / gelatin and NaCl organohydrogel	420 V, 12 μA (55×55 mm^2)	○ (-20 °C)	×	○ (~300 %)	--
80	Polyurethane / polyacrylamide, montmorillonite, and carbon nanotube organohydrogel	86.4 V, 1.1 μA (4 Hz, 3 cm×4 cm)	○ (-60 °C)	×	○ (~4196 %)	--
29	Silicone rubber/ LiCl, graphene oxide, and ethylene glycol	317 V, 27 μA (40×80 mm^2)	○ (-40 °C)	×	○ (200 %)	--
72	Polydimethylsiloxane/ ZnO, acylic acid, and 1-ethyl-3-methylimidazolium dicyanamide	189 V, 6.2 μA (1.5 Hz, 3×3 cm^2)	○ (-20 °C)	○ (98 %, 2 h, rt)	○ (~1000 %)	--
73	2(6-Isocyanatohexylaminocarbonylamino)-6-meth yl-4[1H]pyrimidinone, polydimethylsiloxane / acrylamide glycerol, and N,N,N',N'-tetramethylethylenediamine hydrogel	157 V, 16 μA (4×4 cm^2)	○ (-30 °C)	○ (~92 %)	○ (~3360 %)	--
74	VHB / Polyacrylamides, nano-clays, ethylene glycol/water	86 V, 0.76 μA (2.5 Hz, 3×3 cm^2)	○ (-30 °C)	○	○ (1124 %)	--
75	Silicone rubber/ alpha-lipoic acid, phytic acid, Fe^{3+}, PEDOT: PSS organohydrogel	~47 V, 370 nA (4 Hz, 5×5 cm^2)	○ (-40 °C)	○ (4 min / 24 h)	○ (5000 %)	--

Table S3. Comparative articles of anti-freezing TENGs.

Details for other supplementary materials

Movie 1

Demonstration of the SDDE-TENG harvesting biomechanical energy to drive a smart watch.

Movie 2

Demonstration of the SDDE-TENG harvesting biomechanical energy to drive a smart watch after cutting and self-healing.

Movie 3

Demonstration of the SDDE-TENG-based self-powered human-machine interface device to operate the classical computer game ‘‘Snake Game’’.

Movie 4

Demonstration of the SDDE-TENG self-powered human-machine interface device to operate the classical computer game ‘‘Snake Game’’ after cutting and self-healing.

Movie 5

Demonstration of the SDDE-TENG harvesting biomechanical energy to light 48 parallel LEDs in a snowfield.

Movie 6

Demonstration of the SDDE-TENG harvesting biomechanical energy to light 48 parallel LEDs in a snowfield after cutting and self-healing.
